# A Tailored mHealth Intervention for Improving Antenatal Care Seeking and Health Behavioral Determinants During Pregnancy Among Adolescent Girls and Young Women in South Africa: Development and Protocol for a Pilot Randomized Controlled Trial

**DOI:** 10.2196/43654

**Published:** 2023-09-13

**Authors:** Ronel Sewpaul, Ken Resnicow, Rik Crutzen, Natisha Dukhi, Afzal Ellahebokus, Priscilla Reddy

**Affiliations:** 1 Department of Health Promotion Care and Public Health Research Institute (CAPHRI) Maastricht University Maastricht Netherlands; 2 Public Health, Societies and Belonging Human Sciences Research Council Pretoria South Africa; 3 School of Public Health University of Michigan Ann Arbor, MI United States; 4 ARCH Actuarial Consulting Cape Town South Africa; 5 College of Humanities University of KwaZulu-Natal Durban South Africa

**Keywords:** antenatal care, adolescent girls and young women, AGYW, adolescent pregnancy, mobile health, mHealth, tailoring, motivational interviewing, South Africa, mobile phone

## Abstract

**Background:**

South Africa, a middle-income country, has an adolescent fertility rate far higher than that of high-income countries. Adolescent girls and young women have an increased risk of pregnancy-related complications and lower antenatal appointment attendance rates than older adult pregnant women. Mobile health (mHealth) interventions to improve health behaviors among pregnant adolescent girls and young women in low- and middle-income countries are scarce.

**Objective:**

This paper describes the development and components of an mHealth intervention to improve antenatal appointment attendance and health behavioral determinants among pregnant adolescent girls and young women in South Africa and details the protocol for a pilot randomized controlled trial that evaluated the intervention’s efficacy and user acceptability.

**Methods:**

The intervention, Teen MomConnect, sent tailored motivational behavior change and behavioral reinforcement SMS text messages to participants about antenatal appointment keeping and pregnancy behaviors. The delivery methodology of the intervention was adapted from MomConnect, an mHealth education program for pregnant women in South Africa that has nationwide coverage. In addition, participants received a face-to-face motivational interviewing session delivered by a trained research assistant. Pregnant adolescent girls and young women aged 13 to 20 years were recruited from health facilities and community networks. Participants were randomized into the control group that received the standard MomConnect health SMS text messages or the experimental group that received the Teen MomConnect intervention. Participants completed a baseline questionnaire upon enrollment in the study and a follow-up questionnaire after the end of their pregnancy. The questionnaires assessed demographic characteristics, pregnancy behaviors, and the psychosocial determinants of antenatal appointment attendance (knowledge, attitudes, social support, risk perceptions, self-efficacy, intention, and action planning). Feasibility was assessed using descriptive analyses of acceptability, study implementation processes, and perceived satisfaction with the intervention. The number of appointments attended was obtained from the participants’ clinic records. Appointment attendance was compared between the control and experimental groups, as were awareness of HIV status and the psychosocial determinants of antenatal appointment attendance.

**Results:**

Participant recruitment was conducted from May 2018 to December 2018, and the questionnaire-based data collection was completed by December 2019. Overall, 412 participants were enrolled.

**Conclusions:**

This paper describes the Teen MomConnect intervention to improve antenatal appointment attendance and pregnancy health behaviors among adolescent girls and young women. The results on the intervention’s preliminary efficacy and user acceptability will inform policy makers and health program officers on how tailored, age-appropriate, and motivational health behavior messages can be delivered via mobile phone to pregnant adolescent girls and young women.

**Trial Registration:**

Pan African Clinical Trial Registry (PACTR) PACTR201912734889796; https://pactr.samrc.ac.za/TrialDisplay.aspx?TrialID=9565

**International Registered Report Identifier (IRRID):**

DERR1-10.2196/43654

## Introduction

### Background

South Africa’s adolescent fertility rate of 68 births per 1000 girls aged 15 to 19 years is more than quadruple that of high-income countries [1]. Adolescent girls and young women have an increased risk of pregnancy-related complications, including low birth weight, eclampsia, and preterm birth, compared with older women [2-6]. Furthermore, maternal and infant mortality rates are higher in adolescents than in adult women [5,7]. Pregnant adolescents also face problems such as iron deficiency, inadequate nutrition, and higher engagement in tobacco smoking and alcohol and substance use [4] and have more psychological and social needs than older women [4,8].

The adolescent Institutional Maternal Mortality Ratio in South Africa was 76.9 deaths per 100,000 live births in 2014 to 2016 [9]. The Confidential Enquiries into Maternal Deaths found that almost three-quarters of the deaths among adolescent mothers were from factors such as non–pregnancy-related infections (including HIV or AIDS-related infections, tuberculosis, or pneumonia), hypertension, obstetric hemorrhage, and medical and surgical disorders [9].

Maternal health and pregnancy outcomes among adolescent girls and young women can be improved through appropriate antenatal care (ANC) and practicing healthy behaviors during pregnancy. Behaviors that are recommended for a healthy pregnancy include following a healthy diet; practicing physical activity; taking nutrient and folate supplements; practicing oral hygiene; managing preexisting conditions; and avoiding the use of tobacco, alcohol, and illicit substances [10]. Notably, pregnant adolescents often have poor diet quality, inadequate nutritional knowledge [11], and less knowledge of danger signs [12]. These adolescents typically have less knowledge of what to expect and how to appropriately care for themselves during pregnancy [13,14]. Furthermore, environments of lower socioeconomic status hamper the ability to practice healthy pregnancy behaviors. This is relevant as teenage pregnancies in South Africa are more prevalent in areas of lower socioeconomic status [15].

Early initiation and routine attendance to ANC allows for the prevention, early detection, and management of risk factors and pregnancy-related complications and for the provision of pregnancy-related health promotion [16]. In South Africa, where basic ANC is available at public health facilities at no cost, 77% of pregnant adolescents attend the recommended requisite of at least 4 ANC visits [17]. Studies indicate lower ANC attendance and late ANC booking among adolescents and very young women in South Africa than older women [18,19].

Mobile health (mHealth) interventions are increasingly being used to provide educational, motivational, and behavioral content for a range of issues such as smoking cessation, weight loss, and mental and sexual health [20-23]. SMS text messaging has been previously shown to positively influence the uptake of antenatal visits among pregnant women in low- and middle-income countries (LMICs) [24]. SMS text messaging has the ability to reach large populations with no costs incurred by the recipients. The high mobile phone access in South Africa, a middle-income country, provides opportunities for mHealth behavior change programs to reach large numbers of people and improve health behaviors. Currently, 92% of South Africans own a mobile phone [25], with youth being significant contributors to the mobile phone market. As early as 2012, approximately 70% of high school learners reported having their own mobile phone [26].

The MomConnect program [27], launched by the South African National Department of Health in 2014, is one of the largest mHealth programs worldwide. MomConnect uses mobile phones to register pregnancies in a central database and sends SMS text messages containing maternal health information to pregnant women in the language of their choice throughout the course of their pregnancy until 1 year after giving birth. MomConnect messages are based on maternal health SMS text messages from the Mobile Alliance for Maternal Action in South Africa [28]. Pregnant women are routinely assisted in registering on MomConnect when they attend ANC at health facilities. Between August 2014 and April 2017, over 1.1 million women were successfully registered to MomConnect, corresponding to approximately half of the women attending ANC and 60% of those attending ANC that own a mobile phone [29]. Nearly one-third of registrations occur by the end of the first trimester of pregnancy [29].

Given that the MomConnect messages were designed more for adult pregnant women, we posited that it might be beneficial to adapt the message content for adolescent girls and young women. In light of this, a review of MomConnect registrations found that the odds of opting out of MomConnect were higher for younger women, that is, those aged <25 years [29], thus suggesting a need for retaining younger audiences. In addition, MomConnect sends a fixed set of messages based on the stage of pregnancy. A growing number of mHealth interventions provide tailored messages whereby participants receive content that is matched to a sociodemographic, motivational, behavioral, or personality attribute [30,31]. Therefore, messages that are cognitively, language, age, and context appropriate; motivational in nature; and tailored to the behaviors, attitudes, and health status of pregnant adolescent girls and young women may have the potential to increase uptake of content and induce greater behavior change.

### Objectives

There are currently few mHealth studies on interventions that are specifically targeted at improving health behaviors among pregnant adolescent girls and young women in LMICs [32]. Their behavioral, cognitive, and motivational needs and position in society are distinctly different from those of adult women. Therefore, this study sought to develop a mobile-based intervention targeting pregnant adolescent girls and young women. It adapts the MomConnect methodology. It includes motivational behavior change and behavioral reinforcement messages aimed at improving ANC attendance and its psychosocial determinants during pregnancy, as well as tailored message feedback on behavioral and psychosocial determinants. The intervention, titled Teen MomConnect, would be embedded in an SMS text messaging–based system similar to that of MomConnect. An additional component of the Teen MomConnect intervention is a motivational interviewing (MI) face-to-face counseling session delivered by research assistants. This paper describes the development and components of the intervention as well as the protocol for the pilot 2-arm randomized controlled trial (RCT) to evaluate the intervention’s preliminary efficacy and user acceptability.

## Methods

### Development Process

#### Messaging Content

Formative qualitative research with pregnant adolescents, a review of maternal health education resources, and a review of the existing MomConnect messages provided extensive data that were collectively used to develop and refine the Teen MomConnect intervention messages.

First, key behavioral domains were identified to be included. A multidisciplinary team of researchers in behavioral science, health communication, maternal health, and mHealth and individuals from the technical implementing partner of the MomConnect program reviewed the messages in the existing MomConnect program to categorize them into behavioral domains. To further refine the list of behaviors that place young mothers at risk to be relevant to South African pregnant adolescents, existing maternal health education materials such as the Western Cape Department of Health “Caring for mothers, Caring for you” maternity health education booklet, maternity care guidelines in South Africa, and a review of the literature were used. The data gathered from this work resulted in the selection of 13 behavioral domains for the intervention messages, which are listed in Textbox 1. Health communication specialists from the research team drafted a few examples of intervention messages from each of these behavioral domains and their determinants that were to be pretested.

**List of behavioral domains for the intervention messages.**

- Alcohol
- Appointment keeping
- Attitude toward pregnancy
- Clinic attendance
- General health information
- Illegal drugs
- Nutrition
- Self-care and hygiene
- Smoking
- Social stigma
- Social support
- Sexually transmitted diseases
- Tuberculosis

The formative qualitative research with pregnant adolescent girls and young women comprised 4 individual in-depth interviews and 4 mini focus group discussions of between 2 and 5 participants among a sample of 19 pregnant adolescent girls and young women aged 13 to 19 years who attended ANC services in Cape Town, South Africa [33]. The interviews and focus groups investigated experiences with ANC attendance, perceptions of MomConnect, determinants of appointment attendance, and the environmental and social context of participants. In addition, participants were asked to provide feedback on the initial Teen MomConnect sample messages that were drafted by the research team. The findings from the qualitative research informed the fine-tuning of the messages and the development of new messages. The following paragraphs provide a detailed description of the data gathered and how they were used for the creation of the messages.

Feedback from the participants on the MomConnect program in general was that MomConnect provided valuable information about the baby’s development at various stages of pregnancy. The participants valued that the messages communicated directives to attend appointments, practice healthy behaviors, and prepare for labor admission. They found that being able to read the SMS text messages and make sense of them in their own time was more helpful than being told the information all at once at the health facility. Some participants reported that several MomConnect messages contained information that was not applicable to everyone or that they already knew. They expressed the need for language that is simple, concise, and appropriate for young people and suggested including teenage slang to be more understandable. Participants requested more specific information, such as the kind of food to eat rather than generic advice to eat healthily. The need for information on labor, labor signs, hygiene practices, high blood pressure, and the consequences of not exercising was expressed. Some participants reported that they would like to be checked up on regularly to ensure that they were receiving the messages after registering. The feedback indicated an overall need for a more personalized or tailored messaging approach.

Feedback on the Teen MomConnect draft messages was that participants appreciated the specific and practical examples of behaviors and healthy foods provided within the draft messages. They welcomed message content on topics that they were unaware of, such as explanations of why practicing behaviors such as exercise was important. They reported that some messages provided examples that were impractical in their environments, such as exercise activities that were costly or unavailable in their residential areas. Participants suggested including messages that reminded them of their appointment dates. Findings from the focus groups on factors contributing to antenatal appointment attendance and the social context of participants also informed the development and refinement of the intervention messages.

Stakeholder meetings held with professional officers from the Western Cape Department of Health provided further insights into experiences with the MomConnect program, adolescent access to basic ANC services, local health facility operations, and the recruitment of pregnant adolescent girls and young women from the facilities. This information was used to contextualize the proposed intervention content.

Following the stakeholder meetings and the qualitative research findings on experiences with MomConnect and on the perceptions of the Teen MomConnect draft messages, a full set of SMS text messages was developed. The content and tailoring of the messages were based on techniques for tailoring health communication messages used in previous studies [30,31,34] and were grounded in self-determination theory [35]. Therefore, the messages applied an autonomy-supported tone to foster autonomous motivation for behavior change [35]. As such, the messages used phrases such as “consider to,” “you can choose to,” or “have you been able to” instead of authoritative or definitive phrases such as “you should.” Reflective statements and questions rather than commands were often used, such as “What can you do to remind yourself next time?” for participants who forgot to attend their last appointment. The self-determination theory components of enhancing competence and relatedness were incorporated into the message content by providing practical information on behavioral barriers (eg, “giving up [smoking] even now can still have positive effects for you and your baby”) and using language that was supportive, respectful, and relatable. The messages were restricted to 160 characters to comply with the required length of an SMS text message. The messages were developed in English and translated and back translated into isiXhosa and Afrikaans. The translated messages were checked to ensure that they maintained the tone and intent of the original messages.

The final message library comprised 66 messages in English, Afrikaans, and isiXhosa and covered the 13 behavioral domains. The messages were tailored by pregnancy week and the week in which the participant joined the intervention program (join week). The logic flow allowed users who had missed messages from earlier pregnancy weeks to be sent relevant *catch-up messages* with the content they missed. There were 48 static or 1-way messages that did not require the user to respond and 18 “two-way” tailored message sets that asked the user a question. The 2-way messages allowed participants to respond by typing a letter corresponding to their response option. The user would receive a tailored feedback message depending on their response to the question. For example, the question assessing smoking asked the following—“During the past month (30 days), on how many days did you smoke cigarettes?”—with the following options: “a. 0,” “b. 1 to 15,” and “c. 15 to 30.” If the participant answered “a,” then they would receive the following message: “Well done! By not smoking during pregnancy you will have much lower chances of having a low birth-weight.” If they answered “b” or “c” or did not respond to the question, they would receive the following message: “Quitting smoking can be hard but it is one of the most important things you can do for a healthy pregnancy and healthy delivery.” Therefore, the logic flow was designed to send a customized response even when the user did not respond to the message. In this way, the intervention would allow each user to receive a unique set of messages based on their pregnancy week and responses to questions. With regard to appointment attendance behavior, the logic flow applied iterative tailoring for 2-way messages on appointment attendance. These included appointment reminders every 4 weeks for those who did and did not know their appointment dates, checkups of whether the appointments were actually attended, and tailored feedback addressing various reasons for not attending each appointment. For affirmative responses to 2-way messages on engaging in smoking, alcohol use, and drug use, users would receive tailored follow-up messages every 4 weeks motivating them to stop these behaviors. Examples of messages on appointment attendance and smoking behavior and their logic flows and timing are presented in Multimedia Appendix 1, whereas brief example messages of the remaining 11 message domains are presented in Table 1.

The MomConnect program used an open-source SMS text messaging platform to distribute the messages, and user registrations occurred via Unstructured Supplementary Service Data (USSD). USSD is a communication protocol for mobile phones to communicate with mobile network operators’ computers [29]. The Teen MomConnect intervention messages were delivered via SMS text messages using the same technology platform and user registration process as MomConnect to facilitate comparability. Although a smartphone app was considered to deliver the intervention, SMS text messages were chosen as many adolescents and young people did not have access to a smartphone and an app would incur data costs to users. The intervention messages and the corresponding coding and logic flows were integrated into the technology platform used to implement MomConnect.

**Table 1:** Extract of SMS text messages.

Message domain	Message	Type
Alcohol	It can be hard to give up drinking but it is one of the most important things you can do for a healthy pregnancy and healthy delivery.	Tailored response to consuming alcohol in the past month
Attitude toward pregnancy	We understand pregnancy can be stressful. Many girls feel the same! You can talk to someone you trust about your feelings, or come to a clinic for support.	Tailored response to feeling stressed about their pregnancy
Clinic attendance	Regular check ups at the clinic will ensure that you and your baby stay healthy. Consider attending all your scheduled clinic appointments.	Static
General health information	Your baby’s lungs are starting to develop and its heart beat can be heard.	Static
Illegal drugs	Have you been able to give up hard drugs since you knew that you were pregnant? Reply with a or b. Yes No	Tailored question
Nutrition	Including iron-rich food like eggs, beans, green vegetables and meat will ensure that your baby develops normally.	Static
Self-care and hygiene	Proper hygiene is important to prevent you from getting sick. Regularly wash your hands with soap, especially before eating and after going to the toilet.	Static
Social stigma	Not everyone is kind to young mothers. We know some people may judge you. Talk to someone you trust, or you can speak to a counsellor at the clinic.	Static
Social support	Emotional support can help with moms’ mental and physical health. Think about who you can trust and talk to during this time.	Tailored response to preference for emotional support over informational support
STDs^a^	Finding out about your HIV status can help to make sure you and your baby receive the right medicine on time. You can choose to do an HIV test at your clinic.	Tailored response to unknown HIV status
Tuberculosis	Getting treated can reduce the risk to you and your baby. If you don’t have medicines, talk to your health care worker.	Tailored response to positive tuberculosis test

^a^STD: sexually transmitted disease.

#### Development of the MI Content

The “Difficulty by Motivation” matrix for behaviors and interventions [36] guided the decision to supplement the messaging component with MI. Although tailored messaging can help change behaviors such as attending an appointment, complex behaviors where individuals show higher resistance to changing and require greater motivation and competence to change, such as sustained appointment adherence and substance abuse, often require more intense and interpersonal interventions such as MI. MI [37,38] is an evidence-based, patient-centered style of counseling that has been used in a range of health behavioral interventions in adolescents and adults. MI has been successful in improving health behaviors in pregnant women [39]. The aim of the MI session was to motivate and encourage the participants to attend ANC appointments and improve their health behaviors during pregnancy. MI differs from other types of counseling in that, instead of providing information and directive advice, the participants are encouraged to reflect on and express their own reasons for and against change and how their current behavior affects their life and family goals or core values and explore possible options for making change. Ambivalence and resistance are explored before moving toward action. The development of the MI content was informed by the key themes that emerged from the formative qualitative interviews previously conducted with pregnant adolescent girls and young women; focus group interviews conducted with health care workers and with research assistants who worked with the participants; and an analysis of the behaviors, social norms, and attitudes reported in the baseline questionnaire. The key themes of appointment keeping, substance use, HIV and tuberculosis testing, medication adherence, nutrition, and overall healthy pregnancy behaviors formed the basis of the MI content. The session was tailored to the participants to be relevant to their specific behaviors and questions. The MI sessions were first intended to be conducted by health care workers at the facilities. However, discussions with the facility staff revealed that they could not accommodate delivering these sessions into their already heavy workloads. Research assistants were then identified to conduct the sessions with the participants. The research assistants attended a training workshop facilitated by an expert counselor in MI. The training session taught reflective listening, asking open-ended questions, formulating affirmations, reflective listening, promoting change talk, and developing an action plan to improve behaviors identified as problematic. The research assistants were taught how to balance the expression of empathy with the need to build room for change. One face-to-face MI session was to be delivered by a research assistant during the participants’ pregnancies.

### Study Design

The aims of the study were to evaluate the feasibility of the Teen MomConnect intervention and test its preliminary efficacy on antenatal appointment attendance, health behaviors, and psychosocial mediators.

A pilot 2-arm RCT was conducted. Participants were randomized into the intervention or experimental group that received the Teen MomConnect intervention or the control group that received the standard MomConnect mobile messaging. This study was registered in the Pan African Clinical Trial Registry as trial PACTR201912734889796. This study was conducted in Cape Town in the Western Cape province of South Africa from April 2018 to December 2019. Cape Town is predominantly urban, and 9.5% of its 67,485 in-facility deliveries are among adolescents [40]. In the Western Cape province, 29% of pregnant women present late for their first antenatal appointment, that is, beyond 20 weeks of pregnancy. The overall Institutional Maternal Mortality Ratio in the Western Cape is approximately 50 deaths per 100,000 live births, which is lower than the national average. The Western Cape has the second highest provincial percentage of low–birth weight babies (17.3%) and the lowest provincial proportion of stillbirths (16.6%) [41]. In addition, substance use during pregnancy is high in Cape Town, where 9% of women attending ANC use illicit drugs and one-fifth consume alcohol during pregnancy [42].

A sample of 200 (100 participants/group) was decided upon for the pilot RCT. With this sample, it was determined that there would be sufficient power (≥0.80) to detect a Cohen *d* effect size of ≥0.40. This equates to a difference of 0.52 visits between groups at posttest measurement. This assumes a posttest mean of 4.1 (SD 1.3) visits in the intervention group and 3.58 (SD 1.3) visits in the control group (Cohen *d* = 0.52 / 1.3 = 0.40). Owing to the high expected attrition rate in adolescent longitudinal studies and the fact that participants with missing contact details and pregnancy characteristics would be excluded from registration to the mobile intervention, it was decided to recruit as many participants as possible for the baseline survey.

### Participants

In South Africa, pregnant girls and women receive ANC and maternity services at outpatient clinics, community health centers, or midwife obstetric units (MOUs) within the primary health care system, which serves over 70% of the population. Pregnant adolescent girls and young women aged 13 to 20 years who had access to a mobile phone were eligible to be recruited to participate in the study. The Western Cape Department of Health provided information on priority communities and clinics where youth pregnancy rates were high and provided guidance on recruitment procedures. On the basis of these discussions, 16 community facilities that provided ANC (comprising public health clinics, community health centers, and MOUs) were identified for recruitment. The facilities were located in 4 of the 8 health subdistricts in Cape Town: Cape Town Eastern, Cape Town Northern, Mitchells Plain, and Tygerberg. Participants were recruited while attending ANC at these facilities. The research team discussed recruitment and data collection activities with the facility managers. Research assistants introduced the study to attendees in the antenatal service waiting areas. In some cases, facility staff referred the research assistants to potential participants. Participants were also recruited from communities via social networks. The research assistants explained the study to potential participants in their language of choice. The research assistants were fluent in English and either Afrikaans or isiXhosa, which are the 3 predominant languages spoken in Cape Town.

The study initially aimed to recruit participants who were up to 24 weeks pregnant to maximize their exposure to the messaging content over the duration of their pregnancies. However, a considerable number of pregnant adolescent girls and young women were beyond 24 weeks when approached during recruitment. Therefore, it was decided to recruit all pregnant adolescent girls and young women.

### Recruitment Procedure

A total of 20 trained research assistants conducted recruitment and data collection activities. Recruited participants received information sheets that described the objectives, benefits, and potential risks of the study; the dissemination of findings; the privacy and confidentiality of the study; and their right to withdraw from the study at any time. The participants were asked to complete a consent form (for participants aged ≥18 years) or assent form (for participants aged <18 years) before being enrolled in the study. In addition, parental consent forms were required for participants aged <18 years. Consenting individuals completed a self-administered baseline questionnaire on an electronic tablet or mobile phone. Baseline questionnaire completion was facilitated by the research assistants. In a few cases in which the participant was not comfortable with completing the questionnaire themselves, the research assistant administered the questionnaire. The questionnaire took approximately an hour to complete. Participants received a US $3 incentive for questionnaire completion.

User registration to receive the MomConnect and Teen MomConnect messages required information to be entered on the name, mobile phone number, age, language, and expected date of delivery (EDD) of users. These variables, as well as the estimated last menstrual date (LMD) and current enrollment in MomConnect, were extracted from the baseline data. In the few cases in which the participant did not have their own mobile phone, they were advised to enter into the baseline survey the mobile phone number of someone whom they lived with and whose mobile phone they could use to access the messages sent during the study. The EDD was required as messages are dependent on pregnancy week. In cases in which the EDD was missing, an estimate was calculated using the reported LMD. In cases in which both dates were missing, the participant was contacted telephonically several times to try to obtain either their EDD or LMD. The language was asked to assess the language in which participants would receive their messages. The options were English, Afrikaans, and isiXhosa. Participants were excluded from enrollment in the intervention study if one or more of their EDD, language, name, age, and mobile phone number was missing or if they were already registered on MomConnect.

**Figure 1:**
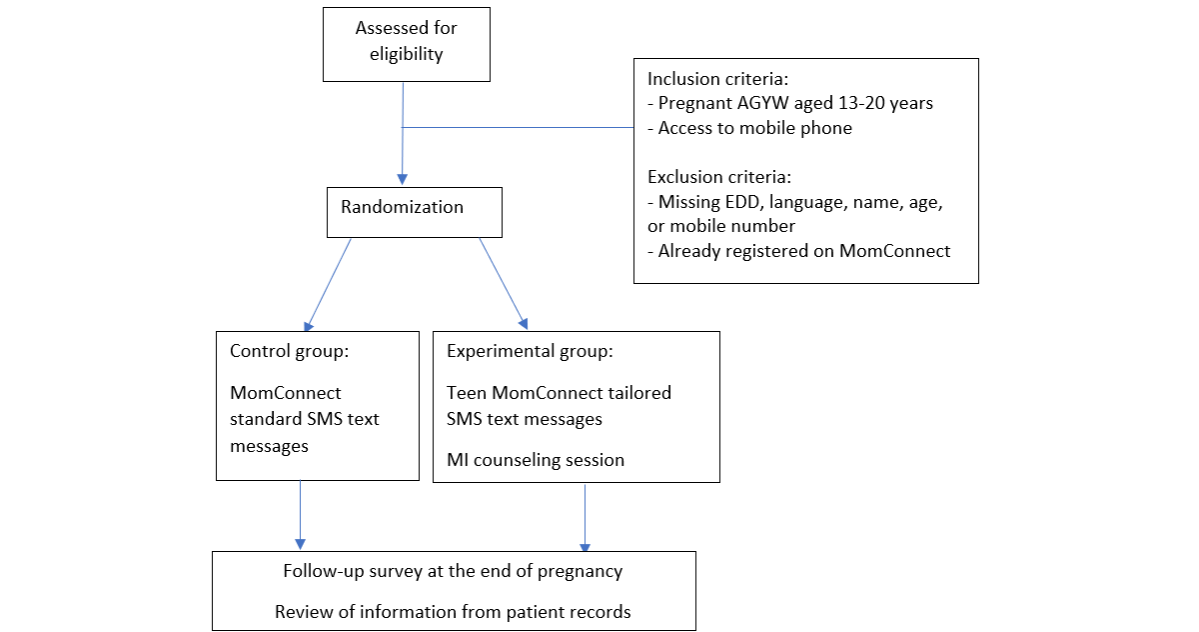
Diagram of the study procedure. AGYW: adolescent girls and young women; EDD: expected date of delivery; MI: motivational interviewing.

Participants were then randomized to the experimental or control group using simple randomization (Figure 1). The study was blinded for participants as they were not told which group they were entered into. Participants were registered on the respective Teen MomConnect and MomConnect messaging programs in weekly or biweekly batches. The control group, which was registered in the MomConnect messaging program, received the MomConnect standard messages according to their pregnancy week and join week. The experimental group participants, who were registered in the Teen MomConnect messaging program, received the messages from the Teen MomConnect SMS text message library based on their pregnancy week, join week, and responses to the tailored questions. In addition, the experimental Teen MomConnect group were to receive 1 face-to-face behavioral counseling group session using MI administered by a trained research assistant.

The research assistants contacted participants from September 2018 to December 2018 to schedule MI sessions at times that were convenient for them. Preliminary interview sessions revealed that some participants did not answer their phones, many scheduled sessions but did not attend them, many could not make time to attend a session because of school and other commitments, and it was difficult to allocate participants to times when all group members were available. Therefore, based on the ascertained limited availability of participants between enrollment into the study and the end of their pregnancy, it was decided to conduct motivational interviews with participants until 30% of the sample had received an interview session. Interview sessions could be conducted one-on-one or in groups of up to 5 people. Participants received an incentive of US $6 for attending a session.

Once the EDD had passed, participants in both the experimental and control groups were programmed to receive the standard postpartum messages that users receive in the MomConnect program. Participants were contacted a week after their EDD had passed to schedule voluntary completion of the follow-up questionnaire. Participants who had an abortion, miscarriage, or stillbirth were also contacted. As with the baseline questionnaire, participants completed the follow-up questionnaire on an electronic device facilitated by the research assistants, and they received a US $3 incentive. Electronic administration of both the baseline and follow-up surveys was facilitated using the Qualtrics electronic survey tool (Qualtrics International Inc) [43].

### Ethics Approval

The Research Ethics Committee of the Human Sciences Research Council (REC 2/17/08/16) granted ethics approval for this study. Permissions were obtained from the Western Cape Provincial Government and the Department of Health to conduct research within the communities’ public health clinics and MOUs.

### Measurement Instruments

Data were gathered from the baseline and follow-up questionnaires and from a review of patient clinical records at the facilities.

#### Questionnaires

Questionnaire development was informed by the reasoned action approach [44] and the Integrated Behavior Change Model (I-Change Model) [45], as well as a literature review on the psychosocial and socioeconomic factors associated with antenatal appointment attendance and health risk behaviors among young pregnant women.

The baseline questionnaire (Multimedia Appendix 2) assessed demographic characteristics; previous pregnancies; attitudes about the pregnancy; mental health status; knowledge of HIV and tuberculosis; HIV and tuberculosis testing; pregnancy risk behaviors (smoking, alcohol use, and illicit drug use); exposure to violence and harassment; perceptions regarding healthy pregnancy behaviors; knowledge regarding appointment attendance; risk perceptions; social support; peer norms; peer, partner, and family attitudes regarding appointment attendance; and participant attitudes, self-efficacy, action planning, and intention toward attending antenatal appointments.

The items on perceptions of healthy pregnancy behaviors, risk perceptions, social support, peer norms, attitudes, self-efficacy, action planning, and intention were all measured on a 4- or 5-point Likert scale, with responses ranging from *strongly disagree* to *strongly agree*.

Perceptions of healthy pregnancy behaviors included items on dietary behavior, nutrient intake, and physical activity. The perception statements were extracted from maternity health information booklets, including the Western Cape Department of Health “Caring for mothers, Caring for you” booklet. Exposure to violence was measured using 13 items on the lifetime frequency of exposure to violence or harassment from relatives, partners, teachers, or health care workers. The questions were adapted from the work by Reddy et al [26]. Knowledge of antenatal appointment attendance was measured using 16 statements informed by maternity booklets. Construction of the attitude, self-efficacy, peer norm, and intention items was based on the reasoned action approach by Fishbein and Ajzen [44], and the action planning, risk perception, and social support items were informed by the I-Change Model [45]. Risk perceptions referred to the perceived implications of not attending or missing antenatal appointments and the risks of pregnancy complications. Social support for attending ANC referred to the encouragement received from family, friends, and partners or boyfriends to attend appointments.

The follow-up questionnaire included the same items as the baseline questionnaire and additional items assessing the outcome of the pregnancy. In addition, evaluation questions were included in the follow-up questionnaire that assessed the participants’ experiences with the SMS text messages and the motivational interview content, overall satisfaction with the study, and experiences with the research assistants. Specifically, 21 items evaluated the messaging component by measuring comprehension, tone, appropriateness, and content of the messages; the perceived degree to which the messages motivated behavior change; the frequency of receiving the messages; and whether participants were able to respond to the messages. The items evaluating the MI were administered to those who reported attending a session. A total of 17 items assessed the perceived degree to which the MI session motivated behavior change, with responses on a 5-point Likert scale.

#### Review of Patient Records

Patient records were accessed from the facilities where the participants were attending ANC or where they gave birth. The patient folder contains the maternity case booklet and other related files, in which facility personnel record information from the patient’s antenatal visits, during labor, and after giving birth. Research assistants and the research team captured the following information from the maternity case booklet and its related files: clinical measures such as blood pressure at antenatal visits, dates of antenatal visits by pregnancy week, HIV and sexually transmitted disease testing, clinical measures from the labor process, and birth weight of the infant. The total number of antenatal visits was obtained from the recorded dates of antenatal visits by pregnancy week.

### Primary and Secondary Outcomes

The primary outcome variables for feasibility were the perceived satisfaction and user acceptability of the intervention, whereas the outcome variable for assessing initial preliminary efficacy was the number of antenatal appointments attended. The number of antenatal appointments attended was obtained from the patient records at the facility. A secondary approach was to code the number of antenatal appointments attended into a binary variable for <4 versus ≥4 visits to calculate the proportion of participants who attended the requisite of at least 4 appointments during pregnancy [17]. Secondary outcomes were awareness of HIV status, psychosocial determinants of antenatal appointment attendance (knowledge, risk perceptions, attitudes, self-efficacy, intentions, and action planning), perceptions of healthy pregnancy behaviors, number of intervention messages responded to, and variables evaluating the recruitment and study implementation processes.

### Statistical Analysis

Analyses will be conducted using SPSS (version 27.0; IBM Corp). Variables on perceived satisfaction with and acceptability of the Teen MomConnect messages and MI sessions in the evaluation section of the follow-up questionnaire as well as the number of messages responded to and MI attendance will be used for descriptive analyses to evaluate how the Teen MomConnect intervention was implemented and received. The outcomes of antenatal appointment attendance and its psychosocial determinants in the experimental (Teen MomConnect) and control (MomConnect) groups will be analyzed using linear regression for continuous outcomes and logistic regression for binary outcomes (including the outcome of percentage that attended ≥4 antenatal sessions) at follow-up. Covariates include age, race, previous pregnancies, school attendance, pregnancy week at enrollment, and exposure to MI. For outcomes related to knowledge, risk perceptions, attitudes, self-efficacy, intentions, and action planning scores that were measured at both baseline and follow-up, the change in mean values between baseline and follow-up in the experimental and control groups will be analyzed using analysis of covariance [46]. For each of these outcomes, moderation effects for race, gestational age at enrollment, socioeconomic status, age, and number of previous pregnancies will be tested with interaction terms in the regression models.

### Data Management, Confidentiality, and Privacy Protection

The data from Qualtrics were protected by log-in ID and password. The server that stored data on message sends and responses was password protected. The data were sent to the statistician and principal investigator upon study completion. The consent forms are kept in a locked storeroom.

### Quality Assurance

Practical training on recruitment and data collection procedures, with refresher sessions, was provided to the research assistants throughout the study to ensure its validity and accuracy. The baseline and follow-up data collected were checked for possible errors an average of every 2 weeks. The research assistants encouraged participants to ask questions about any concerns they had with the study. The research team monitored the study to oversee its progress. The research assistants provided feedback to the project manager 3 to 4 times per week on the study progress and any barriers encountered.

## Results

Recruitment of participants was conducted from May 2018 to December 2018. A total of 412 eligible participants were enrolled in this trial (Table 2). The participant characteristics did not differ significantly between the experimental and control groups, with the exception of average gestational week (control group: 23.1, SD 7.69; experimental group: 20.8, SD 6.76; *P*=.002). The mean age was 18.0 (SD 1.56) years; 36.2% (149/412) were attending an educational institution, 75.4% (307/407) lived in formal dwellings, 59.6% (242/406) reported household shortages of important items, and 10.9% (45/412) had been pregnant before. Of the 412 enrolled participants, 256 (62.1%) completed the follow-up questionnaire. Patient records were able to be retrieved for 54.4% (224/412) of the participants. Results on the preliminary efficacy and acceptability of the intervention will be disseminated via publications and conference proceedings throughout 2023.

**Table 2:** Characteristics of the sample at baseline (n=412)^a^.

			Control (n=219)		Intervention (n=193)		Total sample		*P* value^b^	
Age (years), mean (SD)			18.1 (1.49)		17.9 (1.64)		18.0 (1.56)		.21	
Gestational age (weeks), mean (SD)			23.1 (7.69)		20.8 (6.76)		22.0 (7.35)		.002	
**Racial group^c,d^, n (%)**										.29
	Black African	59 (27.2)		64 (33.2)		123 (30)				
	Coloured	157 (72.4)		128 (66.3)		285 (69.5)				
	Other	1 (0.5)		1 (0.5)		2 (0.5)				
**Attending an educational institution, n (%)**										.29
	Do not attend educational institution	145 (66.2)		118 (61.1)		263 (63.8)				
	Attend educational institution	74 (33.8)		75 (38.9)		149 (36.2)				
**Dwelling type^e^, n (%)**										.79
	Informal	54 (25.1)		46 (24)		100 (24.6)				
	Formal	161 (74.9)		146 (76)		307 (75.4)				
**Self-perceived cost of living^f^, n (%)**										.12
	Food and clothes shortage	41 (19.2)		51 (26.6)		92 (22.7)				
	Shortage of other important things	78 (36.5)		72 (37.5)		150 (36.9)				
	Have important basics	95 (44.4)		69 (35.9)		164 (40.4)				
**Had been pregnant before, n (%)**										.73
	Yes	25 (11.4)		20 (10.4)		45 (10.9)				
	No	194 (88.6)		173 (89.6)		367 (89.1)				

^a^Counts do not always sum to 412 due to missing observations for some variables.

^b^Testing the difference in estimates between the control and intervention groups using chi-square tests for categorical variables and 2-tailed *t* tests for continuous variables.

^c^Control group: n=217; total sample: n=410.

^d^Race was self-reported by participants. Racial categories provided in the questionnaire were according to Statistics South Africa’s standard population groups: Black African, Coloured, White, and Indian. The White and Indian categories were collapsed for analysis due to small sample sizes. Race was reported not with the intention of reifying sociocultural constructs but rather to study ongoing health disparities across groups.

^e^Control group: n=215; intervention group: n=192; total sample: n=407.

^f^Control group: n=214; intervention group: n=192; total sample: n=406.

## Discussion

### Synopsis

This paper describes the development of the Teen MomConnect intervention and the protocol for the pilot RCT to measure its feasibility and preliminary efficacy. The intervention aimed to improve antenatal appointment attendance among pregnant adolescent girls and young women using tailored 2-way messaging and MI. The results of the pilot study will inform policy makers and health program officers on how tailored, age-appropriate, and motivational health behavior messages can be delivered via mobile phone to large numbers of young people. The intervention expands the messaging content of the MomConnect program, which already has high coverage, to be relevant to youth. If the intervention shows promising results (eg, in terms of feasibility), it can be tested in a formal, fully powered trial. The findings will also show how the intervention was implemented and inform how it can be improved in the future.

### Implementational Challenges

This study is subject to some foreseen implementational challenges. mHealth interventions often have high dropout rates, especially among young people. This study aimed to mitigate this by allowing for oversampling of baseline participants. This study will assess retention between baseline and follow-up, and the evaluation can inform retention strategies in future mHealth studies with adolescents. Second, many individuals in South Africa frequently change their mobile phone numbers, and this is expected to be common among adolescents and young adults. Studies on the MomConnect program found that messages were not delivered to a large number of users, which was attributed to users having changed their phone numbers [28]. This study was subject to the same problems. Future mHealth programs, particularly those designed for adolescents, can use smartphone apps rather than SMS text messaging–based systems. Nevertheless, SMS text messages were the most practical delivery option for this study as they facilitated comparison with the MomConnect SMS text message delivery and SMS text messages can reach all mobile phone users regardless of whether they have a smartphone and have no added costs to the user. This is particularly relevant in LMICs such as South Africa that experience challenges with mobile data costs, speed, and coverage [47]. Third, capturing information on the number of appointments attended is subject to the availability and ease of accessibility of the patient health records and the accuracy with which this information is captured by personnel at the facility. Therefore, the research team included questions on whether the participants attended all their appointments in the follow-up questionnaire. This question, although subject to self-report bias, can be used to supplement the analyses of appointment attendance.

## Appendix

Multimedia Appendix 1 Extract of SMS text messages on smoking and appointment keeping and the logic for tailoring and timing of delivery.

Multimedia Appendix 2 Baseline questionnaire.

## Abbreviations

ANC: antenatal care
EDD: expected date of delivery
LMD: last menstrual date
LMICs: low- and middle-income countries
mHealth: mobile health
MI: motivational interviewing
MOU: midwife obstetric unit
RCT: randomized controlled trial
USSD: Unstructured Supplementary Service Data

## Data Availability

The data sets generated and analyzed during this study are available from the corresponding author upon reasonable request.
