# Supplementary material for: A Tailored mHealth Intervention for Improving Antenatal Care Seeking and Health Behavioral Determinants During Pregnancy Among Adolescent Girls and Young Women in South Africa: Development and Protocol for a Pilot Randomized Controlled Trial
Source: JMIR Res Protoc. 2023 Sep 13;12:e43654. doi: 10.2196/43654 (PMC10534293; doi:10.2196/43654)
Supplement: Multimedia Appendix 1 [file resprot_v12i1e43654_app1.docx]

**Table 1.** Extract of SMS text messages on smoking and appointment keeping and the logic for tailoring and timing of delivery.

| Message UID^a^ | Type of message | Question/response logic | Delivery timing logic | Message text | Behavioral domain | Fixed or variable timing (catch-up) |
| --- | --- | --- | --- | --- | --- | --- |
| M5 | Tailored question | N/A^b^ | Join day+2 days | During the past month (30 days), on how many days did you smoke cigarettes? Reply with a, b or c.  a. 0  b. 1 to 15  c. 15 to 30 | Smoking | Variable/catch-up |
| M5.1 | Tailored response: user choosing option (a) from M5 | If M5=“a” | Join day+2 days | Well done! By not smoking during pregnancy you will have much lower chances of having a low birth weight baby. | Smoking | Variable/catch-up |
| M5.2 | Tailored response: user choosing option (b) or (c) from M5 or not responding to M5 | If M5 <> “a” or M5="" | Join day+2 days | It can be hard to give up smoking but it is one of the most important things you can do for a healthy pregnancy and healthy delivery. | Smoking | Variable/catch-up |
| M9 | Tailored question | N/A | Pregnancy week=6, 10, 14, 18, 22, 26, 30, and 34 | Do you know when you next clinic appointment is? Reply with a or b.  a. Yes  b. No | Appointment keeping tailored | Fixed |
| M9.1 | Tailored response/question: user choosing (a) from M9 | If M9=“a” | Pregnancy week=6, 10, 14, 18, 22, 26, 30, and 34 | Please choose month from the list below. Reply with a letter from a to l.  a. Jan  b. Feb  c. Mar  d. Apr  e. May  f. Jun  g. Jul  h. Aug  i. Sep  j. Oct  k. Nov  l. Dec | Appointment keeping tailored | Fixed |
| M9.1.1 | Tailored response/question: user choosing (a) from M9 | If M9=“a” | Pregnancy week=6, 10, 14, 18, 22, 26, 30, and 34 | Now, reply with the date from 1 to 31 | Appointment keeping tailored | Fixed |
| M9.2 | Tailored response: user choosing (b) from M9 or not responding to M9 | If M9=“b” or M9="" | If M9=“a,” deliver 1 week before answer to M9.1 and M9.1.1; if M9=“b,” deliver 21 days after enrollment date and every 30 days thereafter until pregnancy week 36 | This is a reminder that you have a clinic appointment coming up shortly. Do you have any questions about your pregnancy for your health worker? | Appointment keeping tailored | Fixed |
| M9.3 | Tailored question: user will receive this message a day after her clinic appointment | N/A | If M9=“a,” deliver 1 week before answer to M9.1 and M9.1.1; if M9=“b,” deliver 21 days after enrollment date and every 30 days thereafter until pregnancy week 36 | Were you able to keep your appointment to attend the clinic? Reply with a or b.  a. Yes  b. No | Appointment keeping tailored | Fixed |
| M9.3.1 | Tailored response: user choosing option (a) from M9.3 | If M9.3=“a” | If M9=“a,” deliver 1 week before answer to M9.1 and M9.1.1; if M9=“b,” deliver 21 days after enrollment date and every 30 days thereafter until pregnancy week 36 | Well done, regular checks will ensure that you and your baby stay healthy throughout your pregnancy. | Appointment keeping tailored | Fixed |
| M9.3.2 | Tailored question: user choosing option (b) from M9.3 or not responding to M9.3 | If M9.3=“b” or M9.3="" | If M9=“a,” deliver 1 week before answer to M9.1 and M9.1.1; if M9=“b,” deliver 21 days after enrollment date and every 30 days thereafter until pregnancy week 36 | Why did you not attend your appointment? Reply with a, b, c, d, or e.  a. I forgot  b. I was sick  c. I did not want to go  d. I was too busy to go  e. Other | Appointment keeping tailored | Fixed |
| M9.3.2.1 | Tailored response: user choosing option (a) from M9.3.2 or not responding to M9.3.2 | If M9.3.2=“a” or M9.3.2="" | If M9=“a,” deliver 1 week before answer to M9.1 and M9.1.1; if M9=“b,” deliver 21 days after enrollment date and every 30 days thereafter until pregnancy week 36 | What can you do to remind yourself next time? Use your phone calendar, set up reminders, little notes or ask someone to remind you... | Appointment keeping tailored | Fixed |
| M9.3.2.2 | Tailored response: user choosing option (b) from M9.3.2 | If M9.3.2=“b” | If M9=“a,” deliver 1 week before answer to M9.1 and M9.1.1; if M9=“b,” deliver 21 days after enrollment date and every 30 days thereafter until pregnancy week 36 | Are you feeling better? Frequent checks will ensure that you and your baby stay healthy. What can you do to make your next visit? | Appointment keeping tailored | Fixed |
| M9.3.2.3 | Tailored response: user choosing option (c) from M9.3.2 | If M9.3.2=“c,” “d,” or “e” | If M9=“a,” deliver 1 week before answer to M9.1 and M9.1.1; if M9=“b,” deliver 21 days after enrollment date and every 30 days thereafter until pregnancy week 36 | Sometimes these visits can be hassle, but going to the clinic is a good opportunity to learn about your baby’s health. What can you do to attend your next visit? | Appointment keeping tailored | Fixed |

^a^UID: unique identifier

^b^N/A: not applicable.
