# Supplementary material for: A Tailored mHealth Intervention for Improving Antenatal Care Seeking and Health Behavioral Determinants During Pregnancy Among Adolescent Girls and Young Women in South Africa: Development and Protocol for a Pilot Randomized Controlled Trial
Source: JMIR Res Protoc. 2023 Sep 13;12:e43654. doi: 10.2196/43654 (PMC10534293; doi:10.2196/43654)
Supplement: Multimedia Appendix 2 [file resprot_v12i1e43654_app2.pdf]

Baseline questionnaire
Teen MomConnect

Survey Flow

Standard: Introduction message (1 Question)
Standard: Introduction and welcome (0 Questions)
Standard: Basic information (4 Questions)
Standard: Section A: Sociodemographic information (21 Questions)
Standard: History of pregnancy (10 Questions)
Standard: Current pregnancy (9 Questions)
Standard: Access to cell phone (6 Questions)
Standard: Tobacco, alcohol and drug use (4 Questions)
Standard: Experiences of violence (2 Questions)
Standard: Healthy lifestyle and includes nutrition, physical activity, HIV, TB and stress (2 Questions)
Standard: Symptoms of Depression (8 Questions)
Standard: HIV and Tuberculosis (TB) (15 Questions)
Standard: Knowledge (1 Question)
Standard: Risk perceptions (2 Questions)
Standard: Attitudes (2 Questions)
Standard: Social influences (2 Questions)
Standard: Peer norms (2 Questions)
Standard: Family norms (2 Questions)
Standard: Partner/boyfriend norms (2 Questions)
Standard: School norms (2 Questions)
Standard: Self-efficacy (2 Questions)
Standard: Intention (2 Questions)
Standard: Action planning (2 Questions)
Block: (0 Questions)

Page Break

Start of Block: Introduction message

A Pilot Study of Improving Outcomes in Teenage Pregnancy Using a Combined Tailored M- Health Program and Motivational Interviewing Intervention
Thank you for participating in this study on improving the outcomes of teenage pregnancy.
We greatly value your participation in this research study and your willingness to share your knowledge and experiences. Some of the information we will ask you may be sensitive which may make you a bit uncomfortable. We would like you to take your time and think about the answers and respond as honestly as possible to assist with this important research.

End of Block: Introduction message

Start of Block: Introduction and welcome

Start of Block: Basic information

Basic information

Q1 Name

____

Q2 Cellphone/telephone number (Kindly use the following format XXXXXXXXXX)

○ Main number (1) ____

○ Alternative number (2) ____

○ Alternative number (3) ____

Q3 Was consent obtained?

▢ Yes (1)

▢ No (2)

Skip To: End of Survey If Was consent obtained? = No

End of Block: Basic information

Start of Block: Section A: Sociodemographic information

Sociodemographic information

Q4 Date of birth (dd/mm/yyyy)

○ Date of birth (1) ____

*

Q5 ID number (Kindly use the following format 0201400635086)

____

*

Q6 Passport number

____

Q7 Race

○ Black African (1)

○ White (2)

○ Coloured (3)

○ Indian/Asian (4)

○ Other, please specify (5) ____

Q8 Nationality

○ South Africa (1)

○ Other, please specify (3) ____

Q9 Are you in school?

○ Yes (1)

○ No (2)

Skip To: Q10 If Are you in school? = Yes
Skip To: Q13 If Are you in school? = Yes
Skip To: Q11 If Are you in school? = No

*

Q10 What grade are you in? (6,7,8,9,10,11,12)

____

Skip To: Q13 If What grade are you in? (6,7,8,9,10,11,12) Is Not Empty
Skip To: Q11 If What grade are you in? (6,7,8,9,10,11,12) Is Empty

Q11 Do you attend a FET college ?

○ Yes (1)

○ No (2)

Skip To: Q12 If Do you attend a FET college ? = Yes
Skip To: Q14 If Do you attend a FET college ? = No

Q12 What are you studying?

____

Q13 In which community is your school located?

____

Q14 Which community do you live in?

____

Q15 Is your pregnancy registered at a clinic?

○ Yes (1)

○ No (2)

Skip To: Q16 If Is your pregnancy registered at a clinic? = Yes
Skip To: Q17 If Is your pregnancy registered at a clinic? = No

Q16 What is the name of the clinic that your pregnancy is registered at?

____

Skip To: Q18 If What is the name of the clinic that your pregnancy is registered at? Is Not Empty

Q17 What is the name of the clinic where you want to register your pregnancy at?

____

Q18 What is the due date of your pregnancy, if known? (dd/mm/yyyy)

○ Due date (1) ____

Q19 What was the date of your last menstrual period? (dd/mm/yyyy)

- ○ Date of last menstrual period (1) ____

Q20 What type of dwelling do you live in?

- ○ Brick house (1)
- ○ Flat / apartment (2)
- ○ A shack (3)
- ○ A house made of mud and stick (4)
- ○ A house made of mud, brick and stick (5)
- ○ Other (6) ____

Q21 Does your father (male guardian) have a paid job? (Paid job also refers to those who are self-employed e.g. your father has a shop at home)

- ○ Yes, works 5 or more days a week (1)
- ○ Yes, works less than 5 days a week (2)
- ○ No, gets a social grant (3)
- ○ No, unemployed (4)
- ○ No, is ill/disabled (5)
- ○ No, is retired (6)
- ○ My father is deceased (7)
- ○ I don’t know (8)

Q22 Does your mother (female guardian) have a paid job? (Paid job also refers to those who are self-employed e.g. your mother has a shop at home)

- Yes, works 5 or more days a week (1)
- Yes, works less than 5 days a week (2)
- No, gets a social grant (3)
- No, unemployed (4)
- No, is ill/disabled (5)
- No, is retired (6)
- My mother is deceased (7)
- I don’t know (8)

Q23 The cost of living is a concern for many families. Can you tell me which option best describes your household’s situation?

- Not enough money for basic things like food and clothes (1)
- Money for food and clothes, but short on many other things (2)
- We have most of the important things, but few luxury goods (3)
- Money for extra things such as holidays and luxury goods (4)

End of Block: Section A: Sociodemographic information

Start of Block: History of pregnancy

History of pregnancy

Q24 Have you been pregnant before?

- Yes (1)
- No (2)

Skip To: Q25 If Have you been pregnant before? = Yes
Skip To: Q26 If Have you been pregnant before? = Yes
Skip To: End of Block If Have you been pregnant before? = No

*

Q25 If yes, how many times?

____

Q26 Did you experience bleeding during pregnancy or after delivery?

- ◯ Yes (1)
- ◯ No (2)

Q27 What kind of delivery did you have?

- ◯ Vaginal delivery (1)
- ◯ Caesarean Section (C-Section) (2)
- ◯ Other, please specify (3) ____

Q28 Was it an assisted delivery? (Vaginal delivery of a baby performed with the help of forceps or a vacuum device)

- ◯ Yes (1)
- ◯ No (4)

Q29 Have you had a stillbirth before?

- ◯ Yes (1)
- ◯ No (3)

Q30 Have you had a miscarriage before?

- ◯ Yes (1)
- ◯ No (3)

Q31 Is your child still alive?

- ◯ Yes (1)
- ◯ No (3)

Q32 Have you been registered on Mom Connect for previous pregnancies?

- ◯ Yes (1)
- ◯ No (2)

End of Block: History of pregnancy

Start of Block: Current pregnancy

Q33 Current pregnancy

Q34 Did you agree to have sex when you became pregnant?

- ◯ Yes (1)
- ◯ No (3)

Q35 What is your relationship to the person that made you pregnant?

- ◯ Boyfriend (1)
- ◯ Neighbour (17)
- ◯ Relative (18)
- ◯ Teacher (19)
- ◯ Friend (20)
- ◯ Classmate (21)
- ◯ Stranger (22)
- ◯ Casual boyfriend (23)
- ◯ Someone who paid you for sex (24)

Q36 Was the person who made you pregnant __________________ older than you?

- ◯ Younger (1)
- ◯ Same age (6)
- ◯ 1- 5 years older (7)
- ◯ 6 -10 years older (8)
- ◯ 11 or more years older (9)

Q37 How do you feel about this pregnancy?

- ○ Happy (1)
- ○ Sad (8)
- ○ Angry (9)
- ○ Afraid (10)
- ○ Embarrassed/Ashamed (11)
- ○ Confused (12)

Q38 Did you consider having an abortion?

- ○ Yes (1)
- ○ No (3)

Skip To: Q39 If Did you consider having an abortion? = Yes
Skip To: Q40 If Did you consider having an abortion? = No

Q39 Where did you have an abortion?

- ○ A Private facility (1)
- ○ A government hospital (5)
- ○ A traditional healer (6)
- ○ A backstreet abortion (7)

Q40 Did anyone ask you to have an abortion?

- ○ Yes (1)
- ○ No (3)

Skip To: Q41 If Did anyone ask you to have an abortion? = Yes
Skip To: End of Block If Did anyone ask you to have an abortion? = No

Q41 Please select all the people who asked you? (Multiple responses possible)

- ▢ Boyfriend (1)
- ▢ Relative/s (such as parent, sister, brother, grandparent, aunts, other relatives) (8)
- ▢ Friend (9)
- ▢ Teacher (10)
- ▢ Neighbour (11)
- ▢ Health care worker (12)
- ▢ No one asked me to have an abortion (13)

End of Block: Current pregnancy

Start of Block: Access to cell phone

Access to cell phone

Q42 Is your cellphone:

- ○ Your own (1)
- ○ I share a cellphone with someone (2)

Q43 Is your cellphone a basic phone (can only send SMS's and receive calls), or is your cellphone a smart phone (can download WhatsApp, BBM, Facebook, and other cell phone applications)? Please select.

- ○ Basic phone (1)
- ○ Smart phone (2)

Q44 What do you use your cellphone for? (you may choose more than one option)

- ☐ I use my cellphone for making and receiving calls (1)
- ☐ I use my cellphone for social networks (e.g. Facebook, WhatsApp, Twitter, BBM, or chat zone services) (7)
- ☐ I use my cellphone for SMSs (8)
- ☐ I use my cellphone for surfing the internet (9)

Q45 Have you been registered on Mom Connect for your current pregnancy?

- ○ Yes (1)
- ○ No (2)

Q46 What language would you prefer to receive your messages?

- ○ English (1)
- ○ Afrikaans (2)
- ○ IsiXhosa (3)

End of Block: Access to cell phone

Start of Block: Tobacco, alcohol and drug use

Tobacco, alcohol and drug use

Q47 During the past month, on how many days did you have at least one drink of alcohol?

- ○ 0 (1)
- ○ 1 to 5 (7)
- ○ 6 to 9 (8)
- ○ 10 to 19 (9)
- ○ e. 20 to 30 (10)

Q48 During the past month (30 days), on how many days did you smoke cigarettes, cigars, pipes, or e-cigarettes, or use smokeless tobacco such as snuff or chewing tobacco?

- 0 (1)
- 1 or 2 (13)
- 3 to 5 (14)
- 6 to 9 (15)
- 10 to 19 (16)
- 20 to 29 (17)
- All 30 (18)

Q49 In the past 30 days, how many times have you used drugs such as weed/dagga(?), tik, ecstasy, or mandrax?

- 0 (1)
- 1 or 2 (8)
- 3 to 9 (9)
- 10 to 19 (10)
- 20 or more (11)

End of Block: Tobacco, alcohol and drug use

Start of Block: Experiences of violence

Experiences of violence

Q50 In your whole lifetime, how many times have you experienced the following?

	1= Never (1)	2=Once or twice (2)	3=A few times (3)	4=Many times (4)
50.1Threat by a relative (1)	○	○	○	○
50.2 Physical violence by a relative (30)	○	○	○	○
50.3 Been touched by a relative in a way that makes you uncomfortable (31)	○	○	○	○
50.4 Threat by a teacher (32)	○	○	○	○
50.5 Physical violence by a teacher (33)	○	○	○	○
50.6 Been touched by a teacher in a way that makes you uncomfortable (34)	○	○	○	○
50.7 Threat by a boyfriend/partner (35)	○	○	○	○
50.8 Physical violence by a boyfriend/partner (36)	○	○	○	○
50.9 Been touched by a boyfriend/partner in a way that makes you uncomfortable (37)	○	○	○	○
50.10 Threat by a health care worker (10)	○	○	○	○
50.11 Physical violence by a health care worker (38)	○	○	○	○
50.12 Been touched by a health care worker in a way that makes you uncomfortable (39)	○	○	○	○
50.13 Been physically forced to have sex (when the penis enters the vagina or anus) when you did not want to (40)	○	○	○	○

End of Block: Experiences of violence

Start of Block: Healthy lifestyle and includes nutrition, physical activity, HIV, TB and stress

Healthy lifestyle and includes nutrition, physical activity, HIV, TB and stress

Q51 This section is about a healthy lifestyle and includes nutrition, physical activity, HIV, TB and stress. You will not be judged based on the way you answer. Please answer to the best of your ability. Please select your response.

	Strongly Disagree (1)	Disagree (2)	Agree (3)	Strongly Agree (4)	I don't know (5)
51.1 Pregnancy is a good time for me to focus on eating correctly (1)	○	○	○	○	○
51.2 I want to eat a healthy balanced diet during pregnancy (49)	○	○	○	○	○
51.3 It is okay to miss meals in a day (50)	○	○	○	○	○
51.4 Healthy snacks between meals are good (51)	○	○	○	○	○
51.5 Healthy eating keeps my energy levels up (52)	○	○	○	○	○
51.6 If I miss meals my baby will still be fine (53)	○	○	○	○	○
51.7 I can eat as much potatoes or bread, as I want in my pregnancy. (54)	○	○	○	○	○
51.8 Foods high in fats and sugar are good for my pregnancy (55)	○	○	○	○	○
51.9 I can drink as much tea/coffee as I want during my pregnancy (56)	○	○	○	○	○
51.10 Multivitamins are important to take during my pregnancy as they contain the important nutrients I need for my body (2)	○	○	○	○	○
51.11 If I eat a lot of green leafy vegetables, I don't need to take an iron supplement. (57)	○	○	○	○	○
51.12 Pregnant women should take folic acid before and during pregnancy (58)	○	○	○	○	○
51.13 Foods rich in iron such as meat, poultry, pork and fish are important during pregnancy (59)	○	○	○	○	○
51.14 Milk, maas and yoghurt are good for me during pregnancy (60)	○	○	○	○	○
51.15 Calcium is important for healthy teeth and bones (61)	○	○	○	○	○
51.16 I can lose weight during my pregnancy (62)	○	○	○	○	○
51.17 It is okay to gain up to 16kgs weight during pregnancy (63)	○	○	○	○	○

51.18 Constipation can be minimised by high fibre intake, water and exercise during pregnancy (64)	○	○	○	○	○
51.19 It is important to walk for a few minutes every day during my pregnancy (3)	○	○	○	○	○
51.20 Activities such as dancing, running, and walking fast are safe to do during pregnancy (65)	○	○	○	○	○
51.21 Activities with rapid movements and contact is safe for me at the end of my pregnancy (66)	○	○	○	○	○

End of Block: Healthy lifestyle and includes nutrition, physical activity, HIV, TB and stress

Start of Block: Symptoms of Depression

Symptoms of Depression

Q52 In the past 6 months, have you ever felt sad or hopeless?

- Yes (1)
- No (2)
- I don’t know (3)

Q53 In the past 6 months, have you had problems concentrating, recalling details, and making decisions?

- Yes (1)
- No (2)
- I don’t know (3)

Q54 In the past 6 months, have you been crying more often than usual?

- Yes (1)
- No (2)
- I don’t know (3)

Q55 In the past 6 months, did you ever seriously consider attempting suicide (that is, take some action to end your life)?

- Yes (1)
- No (2)
- I don’t know (3)

Q56 In the past 6 months, did you make a plan about how you would attempt suicide (that is take some action to end your life)?

- Yes (1)
- No (2)
- I don’t know (3)

Q57 In the past 6 months, how many times did you actually attempt suicide (that is take some action to end your life)?

- 0 times (1)
- 1 times (2)
- 2 or 3 times (3)
- 4 or 5 times (4)
- 6 or more times (5)

Q58 In the past 6 months, if you attempted suicide, did any attempt result in an injury, poisoning, or overdose that had to be treated by a doctor or nurse?

- I did not attempt suicide (that is take some action to end my life) during the past 6 months (1)
- Yes (4)
- No (5)

End of Block: Symptoms of Depression

Start of Block: HIV and Tuberculosis (TB)

HIV and Tuberculosis (TB)

Q59 What is your HIV status?

- HIV + (1)
- HIV – (5)
- Do not know (6)
- Do not want to answer (7)

Skip To: Q60 If What is your HIV status? = HIV +
Skip To: Q61 If What is your HIV status? = HIV +
Skip To: Q62 If What is your HIV status? = HIV –
Skip To: Q62 If What is your HIV status? = Do not know
Skip To: Q62 If What is your HIV status? = Do not want to answer

Q60 When did you find out you were HIV+?

- Before the pregnancy (1)
- During the pregnancy (5)
- Do not know (6)
- Do not want to answer (7)

Q61 Are you currently on anti-retroviral treatment (ART)?

- Yes (1)
- No (5)
- Do not want to answer (6)

Skip To: Q63 If Are you currently on anti-retroviral treatment (ART)? = Yes
Skip To: Q63 If Are you currently on anti-retroviral treatment (ART)? = No
Skip To: Q63 If Are you currently on anti-retroviral treatment (ART)? = Do not want to answer

Q62 How many times have you been tested during your pregnancy for HIV?

- Zero (1)
- Once (5)
- Twice (6)
- Do not know (7)
- Do not want to tell you (8)

Q63 What is your TB status?

- Positive (1)
- Negative (5)
- Do not know (6)
- Do not want to answer (7)

Skip To: Q64 If What is your TB status? = Positive
Skip To: Q65 If What is your TB status? = Negative
Skip To: Q65 If What is your TB status? = Do not know
Skip To: Q65 If What is your TB status? = Do not want to answer

Q64 Are you currently on treatment?

- Yes (1)
- No (2)

Skip To: Q66 If Are you currently on treatment? = Yes
Skip To: Q66 If Are you currently on treatment? = No

Q65 Have you been tested for TB during your pregnancy?

- Yes (1)
- No (2)
- Don’t want to answer (4)

Q66 If you are infected with HIV or with TB, do you think you can infect your new-born baby with TB/HIV if you are not on treatment?

- Yes (1)
- No (5)
- Do not know (6)
- Do not want to answer (7)

Q67 If I am diagnosed with HIV early, the outcome for the baby is good

- ○ Yes (1)
- ○ No (6)
- ○ Do not know (7)
- ○ Do not want to answer (8)
- ○ I do not have HIV (9)

Q68 If I am diagnosed with TB early, the outcome for the baby is good

- ○ Yes (1)
- ○ No (6)
- ○ Do not know (7)
- ○ Do not want to answer (8)
- ○ I do not have TB (9)

Q69 If the diagnosis and treatment for HIV are delayed it can increase the risk of early labour

- ○ Yes (1)
- ○ No (6)
- ○ Do not know (7)
- ○ Do not want to answer (8)

Q70 If the diagnosis and treatment for TB are delayed it can increase the risk of early labour

- ○ Yes (1)
- ○ No (6)
- ○ Do not know (7)
- ○ Do not want to answer (8)

Q71 TB can be cured with special antibiotics, which can be taken safely during pregnancy.

- ○ Yes (1)
- ○ No (6)
- ○ Do not know (7)
- ○ Do not want to answer (8)

Q72 I will be able to breastfeed my baby even if I have TB or HIV

- ○ Yes (1)
- ○ No (5)
- ○ Do not know (6)
- ○ Do not want to answer (7)

End of Block: HIV and Tuberculosis (TB)

Start of Block: Knowledge

Q73 The section is about your current knowledge on antenatal care. Please answer to the best of your ability. Please select your response.

73.1 Antenatal care appointment benefits the baby, not the pregnant woman. (1)	▼ Agree (1) ... I don't know (3)
73.2 There is no need to have the first antenatal appointment booked as early as possible (before 12 weeks of pregnancy), because it can be done at any time during pregnancy. (17)	▼ Agree (1) ... I don't know (3)
73.3 If a pregnant woman is underweight, she needs extra antenatal care. (18)	▼ Agree (1) ... I don't know (3)
73.4 If a pregnant woman is over-weight, she needs extra antenatal care. (19)	▼ Agree (1) ... I don't know (3)
73.5 Pregnant women who do not receive antenatal care are more likely to give birth to a low-weight baby. (20)	▼ Agree (1) ... I don't know (3)
73.6 Pregnant women who do not receive antenatal care are more likely to have miscarriages and give still birth (baby not alive). (21)	▼ Agree (1) ... I don't know (3)
73.7 Pregnant women who receive late antenatal care (after 28 weeks of pregnancy) and their babies, are more likely to have health problems. (22)	▼ Agree (1) ... I don't know (3)
73.8 Preeclampsia is a serious pregnancy problem characterised by raised blood pressure. (23)	▼ Agree (1) ... I don't know (3)
73.9 Antenatal care can prevent maternal mortality (death of a pregnant woman). (24)	▼ Agree (1) ... I don't know (3)
73.10 Antenatal care can prevent child mortality (death of the baby). (25)	▼ Agree (1) ... I don't know (3)
73.11 Attending clinic appointments prepare a pregnant woman to be physically and mentally fit for child birth. (26)	▼ Agree (1) ... I don't know (3)
73.12 Smoking while pregnant is harmful for the development of the unborn baby. (27)	▼ Agree (1) ... I don't know (3)
73.13 Drinking alcohol will affect the health of a pregnant woman's baby (28)	▼ Agree (1) ... I don't know (3)
73.14 Physical exercise is not recommended for pregnant women. (29)	▼ Agree (1) ... I don't know (3)
73.15 If a pregnant woman contracts a sexually transmitted infection and is not treated, it can be transmitted to the baby and cause serious harm. (30)	▼ Agree (1) ... I don't know (3)
73.16 Infants, born to women with untreated TB, may be of lower birth weight. (31)	▼ Agree (1) ... I don't know (3)

End of Block: Knowledge

Start of Block: Risk perceptions

Risk perceptions

Q74 This section is about your perceptions on the clinic visits. Please answer to the best of your ability. Please select your response.

	Strongly Disagree (1)	Disagree (2)	Agree (3)	Strongly Agree (4)	I don't know (5)
74.1 My risk of having pregnancy problems is low. (1)	o	o	o	o	o
74.2 The risk of experiencing preeclampsia (e.g. high blood pressure) is higher, if I don't attend my clinic appointments. (11)	o	o	o	o	o
74.3 The risk of experiencing heavy bleeding during pregnancy or childbirth is higher, if I don't attend my clinic appointments. (12)	o	o	o	o	o
74.4 I think pregnancy problems can develop into something serious and life threatening. (13)	o	o	o	o	o
74.5 Compared to other pregnant teenagers, I am less likely to suffer from complications of pregnancy (e.g. hypertension, preeclampsia, or heavy bleeding during childbirth). (14)	o	o	o	o	o
74.6 Attending clinic visits is important for me (15)	o	o	o	o	o
74.7 Attending clinic visits is hard for me (16)	o	o	o	o	o
74.8 Missing my clinic appointment ONCE will not affect my pregnancy. (17)	o	o	o	o	o
74.9 Attending clinic visits is an inconvenience for me (18)	o	o	o	o	o
74.10 Missing my clinic appointment more than TWICE will affect my pregnancy. (19)	o	o	o	o	o

End of Block: Risk perceptions

Start of Block: Attitudes

Attitudes

Q75 This section is to understand your attitudes about going to clinic appointments. Please answer to the best of your ability. Please select your response.

	Strongly Disagree (1)	Disagree (2)	Agree (3)	Strongly Agree (4)	I don't know (5)
75.1 I think it is important for me to attend ALL the clinic appointments that are arranged for me. (1)	○	○	○	○	○
75.2 I think it is okay/fine for me to miss/skip SOME of the clinic appointments that are arranged for me. (16)	○	○	○	○	○
75.3 Going to clinic appointments will help me understand if my pregnancy is progressing well. (17)	○	○	○	○	○
75.4 Going to clinic appointments will help me detect any potential health problems of my pregnancy. (18)	○	○	○	○	○
75.5 Going to clinic appointment helps me keep track of my baby's health and development. (19)	○	○	○	○	○
75.6 Going to clinic appointments helps me keep track of my own health. (20)	○	○	○	○	○
75.7 Going to clinic appointment will help me detect any health problems with me and my unborn baby early. (21)	○	○	○	○	○
75.8 Going to clinic appointment is helpful because I can find out useful information about my pregnancy. (22)	○	○	○	○	○
75.9 Going to clinic appointment is a waste of time, because it usually takes a long time and it is costly. (23)	○	○	○	○	○
75. 10 I do NOT want to attend my clinic appointments because the health care workers make me afraid by shouting at me. (24)	○	○	○	○	○
75.11 I do NOT want to attend my clinic appointments because the health care workers make it clear that I am not welcomed at the clinic by being hostile. (25)	○	○	○	○	○
75.12 I do NOT want to attend my clinic appointments because I feel that I am being judged and discriminated against by the health care workers. (26)	○	○	○	○	○
75.13 I do NOT want attend my clinic appointments because I am afraid that other people might find out about my pregnancy. (27)	○	○	○	○	○

End of Block: Attitudes

Start of Block: Social influences

Social influences

Q76 This section is about the social support for your pregnancy. Please answer to the best of your ability. Please select your response

	Strongly disagree (1)	Disagree (2)	Agree (3)	Strongly agree (4)	Not applicable (5)
76.1 My family encourages me to go to clinic appointments. (1)	○	○	○	○	○
76.2 My school teachers encourage me to go to clinic appointments. (6)	○	○	○	○	○
76.3 My friends encourage me to go to clinic appointments. (7)	○	○	○	○	○
76.4 My boyfriend/partner/father of child encourages me to go to clinic appointments. (8)	○	○	○	○	○

End of Block: Social influences

Start of Block: Peer norms

Peer norms

Q77 This section is about your friends' behaviours and attitudes of pregnancy. Please answer to the best of your ability. If you do NOT have a friend who has been pregnant, please select 'not applicable'

	Strongly disagree (1)	Disagree (2)	Agree (3)	Strongly Agree (4)	Not applicable (5)
77.1 My friends who are/have been pregnant go to the clinic on the day of their appointment. (1)	○	○	○	○	○
77.2 My friends, who are/ have been pregnant think that it is only necessary to go to the clinic at the end of the pregnancy. (27)	○	○	○	○	○
77.3 My friends, who are /have been pregnant think that if I go to my clinic appointment, I will get helpful advice for maintaining a healthy pregnancy. (28)	○	○	○	○	○
77.4 My friends, who are/ have been pregnant think that the health care workers at the clinic are unfriendly, threatening and rude. (29)	○	○	○	○	○
77.5 My friends, who are/ have been pregnant think that the health care workers at the clinic give them information that is confusing. (30)	○	○	○	○	○
77.6 My frineds, who are /have been pregnant think that the health care workers will prepare me for a safe delivery (31)	○	○	○	○	○

End of Block: Peer norms

Start of Block: Family norms

Family norms

Q78 This section is about your family members' attitudes of your pregnancy. Please answer to the best of your ability. Please select your response.

	Strongly Disagree (1)	Disagree (2)	Agree (3)	Strongly Agree (4)	I don't know (5)
78.1 My family members feels that if I go to my clinic appointment, It is only necessary for me to go at the end of the pregnancy. (1)	○	○	○	○	○
78.2 My family members feel that I do not need to go to the clinic but take traditional pregnancy medicine (13)	○	○	○	○	○
78. 3 My family members feels that it is helpful for me to get correct information about my pregnancy. (14)	○	○	○	○	○
78.4 My family members feels that it is helpful for me to learn about my baby's health and development. (15)	○	○	○	○	○
78.5 My family members feels that I will receive good advice and health care from the health care workers. (16)	○	○	○	○	○
78.6 My family members feels that It will prepare me for a safe delivery (17)	○	○	○	○	○
78.7 My family members feels that I will embarrass them and bring shame to the family. (18)	○	○	○	○	○

End of Block: Family norms

Start of Block: Partner/boyfriend norms

Partner/boyfriend norms

Q79 This section is about your partner/boyfriend's attitudes of pregnancy. Please answer as honestly as you can, and select the number that is appropriate for you. If you do NOT have a partner/boyfriend, please circle "not applicable".

	Strongly disagree (1)	Disagree (2)	Agree (3)	Strongly Agree (4)	Not applicable (5)
79.1 My boyfriend/partner feels that it is only necessary for me to go to the clinic at the end of the pregnancy. (1)	○	○	○	○	○
79.2 My boyfriend/partner feels that if I go to my clinic appointment, it is helpful for me to get correct information about my pregnancy. (11)	○	○	○	○	○
79.3 My boyfriend/partner feels that if I go to my clinic appointment, it is helpful for me to learn about my baby's health and development. (12)	○	○	○	○	○
79.4 My boyfriend/partner feels that if I go to my clinic appointment, I will receive good advice and health care from the health care workers. (13)	○	○	○	○	○
79.5 My boyfriend/partner feels that if I go for an HIV test that I don't trust and love him (14)	○	○	○	○	○
79.6 My boyfriend/partner feels that if I have had an HIV test he does not have to have one (15)	○	○	○	○	○
79.7 My boyfriend/partner feels that since I am already pregnant, we don't have to use a condom when we have sex. (16)	○	○	○	○	○
79.8 Now that I am pregnant, I am worried that my partner/boyfriend will sleep with other girls. (17)	○	○	○	○	○

End of Block: Partner/boyfriend norms

Start of Block: School norms

School norms

Q80 This section is about your school teachers' attitudes of your pregnancy. Please answer to the best of your ability and select the number that is appropriate for you. If you do NOT attend school, or if you have not talked about your pregnancy with your teacher/s, then please tick "not applicable".

	Strongly Disagree (1)	Disagree (2)	Agree (3)	Strongly Agree (4)	Not applicable (5)
80.1 My school teachers think that it is only necessary for me to go to the clinic at the end of the pregnancy. (1)	○	○	○	○	○
80.2 My school teachers think that if I go to my clinic appointment, it is helpful for me to get correct information about my pregnancy. (10)	○	○	○	○	○
80.3 My school teachers think that if I go to my clinic appointment, it is helpful for me to learn about my baby's health and development. (11)	○	○	○	○	○
80.4 My school teachers think that If I go to my clinic appointment, I will receive good advice and health care from the health care workers. (12)	○	○	○	○	○
80.5 My school teachers feel very uncomfortable that I am pregnant and at school. (13)	○	○	○	○	○
80.6 My school teachers feel that I am an embarrassment to the school and a bad influence on the other learners. (14)	○	○	○	○	○

End of Block: School norms

Start of Block: Self-efficacy

Self-efficacy

Q81 This section is about your feelings of confidence in attending the clinic appointments. Please answer to the best of your ability. Please select your response.

	Strongly Disagree (1)	Disagree (2)	Agree (3)	Strongly Agree (4)	Not applicable (5)
81.1 I am confident in my ability to attend clinic appointments, even if the clinic is far from my home. (1)	○	○	○	○	○
81.2 I am confident in my ability to attend clinic appointments, even if I am feeling ill/sick. (10)	○	○	○	○	○
81.3 I am confident in my ability to attend my clinic appointments, when I am feeling lazy and tired. (11)	○	○	○	○	○
81.4 I am confident in my ability to attend my clinic appointments, even if I am embarrassed or ashamed to go to the clinic. (12)	○	○	○	○	○
81.5 I am confident in my ability to attend my clinic appointments, even if I am afraid of the health care workers. (13)	○	○	○	○	○
81.6 I am confident in my ability to attend my clinic appointments, even if the health care workers will gossip about my pregnancy. (14)	○	○	○	○	○
81.7 I am confident in my ability to attend my clinic appointments, even if my family, teachers, boyfriend/partner will not allow me to go (15)	○	○	○	○	○
81.8 I am confident in my ability to attend my clinic appointments, even if other patients will gossip, make judgemental remarks about my being pregnant. (16)	○	○	○	○	○

End of Block: Self-efficacy

Start of Block: Intention

Intention

Q82 This section is about your intention related to clinic appointments. Please answer to the best of your ability. Please select your response.

	Strongly Disagree (1)	Disagree (2)	Agree (3)	Strongly Agree (4)
82.1 I intend to attend my NEXT clinic appointment. (1)	○	○	○	○
82.2 I intend to attend ALL the clinic appointments. (7)	○	○	○	○
82.3 I intend to take the health advice given by the maternal health care workers (e.g. nurses, doctors, counsellors, pharmacists, health promoters) seriously, and use it. (8)	○	○	○	○
82.4 I intend to be honest about everything that the maternal health care workers ask me. (9)	○	○	○	○
82.5 I intend to ask questions if I don't understand something at my clinic appointment. (10)	○	○	○	○

End of Block: Intention

Start of Block: Action planning

Action planning

Q83 This section is your plans related to clinic appointments. Please answer to the best of your ability. Please select your response.

	Strongly Disagree (1)	Disagree (2)	Agree (3)	Strongly Agree (4)	Not Applicable (5)
83.1 I have made/going to make a plan to remind myself to attend my next clinic appointment so that I don't forget. (1)	○	○	○	○	○
83.2 I have made/going to make a plan to travel (walk/take a taxi/ask someone for a lift) to the facility where my clinic appointment is arranged. (5)	○	○	○	○	○
83.3 If I miss my clinic appointment, I will make a plan to reschedule so I do not miss any appointment. (6)	○	○	○	○	○
83.4 If I miss an appointment, I will wait until the next one. (7)	○	○	○	○	○

End of Block: Action planning
